# E-Cigarette Directory Laws, Sales, and Product Availability From 3 Early-Adopting US States

**DOI:** 10.1001/jamanetworkopen.2026.33874

**Published:** 2026-09-15

**Authors:** Fatma Romeh M. Ali, Margaret Mahoney, Elizabeth L. Seaman Jones, Michael A. Tynan, Kristy Marynak

**Affiliations:** 1 CDC Foundation, Atlanta, Georgia

## Abstract

**Importance:**

Flavored e-cigarettes are the most-used tobacco product among US youths and young adults. The US Food and Drug Administration (FDA) has authorized only 45 e-cigarette products, while thousands remain under review. Some states have enacted laws permitting sales of non–FDA-authorized products listed on state directories, but the impact of these laws is unknown.

**Objectives:**

To evaluate the association of directory laws with e-cigarette sales and product availability over time in Alabama, Oklahoma, and Louisiana, the first states to enact these laws.

**Design, Setting, and Participants:**

This cross-sectional study applied a synthetic difference-in-differences approach to assess state-level e-cigarette retail sales from the 4-week period ending January 31, 2021, through the 4-week period ending April 20, 2025. Alabama, Oklahoma, and Louisiana were compared with synthetic controls constructed from 31 states without directories, assessing changes before and after directory publication. Data were analyzed August 1, 2025, to July 20, 2026.

**Exposure:**

Implementation of directory laws.

**Main Outcomes and Measures:**

The primary outcome consisted of per capita e-cigarette nicotine sales, calculated as units sold × e-liquid volume × nicotine concentration, summed across products per state-month and divided by state population. Product availability was a secondary outcome, defined as the number of distinct products sold monthly in each state. Analyses were conducted overall and stratified by flavor and product type.

**Results:**

A total of 1904 state-month observations were included in the analysis. No associations of e-cigarette nicotine sales or product availability with directory listings were observed in Alabama or Oklahoma, where flavored disposables remained dominant. In Louisiana, the law was associated with a significant reduction in sales relative to synthetic controls (average treatment effect [ATT], −38.29% [95% CI, −53.10% to −18.80%). However, event-study analyses showed this decline was temporary, with the estimated treatment effect equal to −19.46% (95% CI, −27.32% to −10.76%) immediately after publication and reaching −63.43% (95% CI, −74.32% to −47.92%) in July 2024, 8 months after implementation. Sales then rebounded and the decrease became statistically nonsignificant by October 2024 as unlisted flavored disposables and menthol cartridges drove sales. In contrast, product availability in Louisiana declined persistently with an ATT of −53.55% (95% CI, −65.67% to −37.14%). In all states, more than half of sales were for unlisted products by the end of the study period.

**Conclusions and Relevance:**

In this cross-sectional study, directory laws in early-adopter states did not produce lasting declines in flavored e-cigarette sales. These findings suggest that directory laws instead might divert resources from evidence-based tobacco control strategies.

## Key Points

**Question:**

How are the first e-cigarette directory laws associated with e-cigarette nicotine sales and product availability over time?

**Findings:**

In this cross-sectional study of 1904 state-month observations, no associations were observed in Alabama or Oklahoma, whereas in Louisiana, sales declined initially but rebounded within 8 months of implementation, driven by flavored disposable e-cigarettes not in the directory and menthol-prefilled cartridges. In contrast, Louisiana’s product availability declined persistently for an average treatment effect of 53.55%.

**Meaning:**

These findings suggest that directory laws in early-adopting states are not associated with lasting declines in sales of flavored e-cigarettes not authorized by the US Food and Drug Administration, which are popular among youths, and may divert resources from evidence-based tobacco control strategies.

## Teaser

This cross-sectional study uses retail scanner data and a synthetic difference-in-differences model to evaluate whether directory laws in Alabama, Oklahoma, and Louisiana are associated with changes in e-cigarette nicotine sales and product availability relative to states without such policies.

## Introduction

E-cigarettes remain the most commonly used tobacco product among US youths, with more than 1.44 million middle and high school students reporting current use in 2025.^1^ Use is also highest among young adults aged 21 to 24 years,^2^ most of whom have never smoked cigarettes.^3^ Notably, more than 89% of youths^1^ and young adults^4^ who use e-cigarettes report using flavored products, with fruit, candy, and dessert flavors being particularly prevalent and a major driver of use.

New tobacco products must receive US Food and Drug Administration (FDA) premarket authorization to be legally marketed.^5^ E-cigarette manufacturers with products on the market as of August 8, 2016, could continue sales for 1 year if they submitted Premarket Tobacco Product Applications (PMTAs) by September 9, 2020.^6^ As of June 2026, the FDA had authorized 45 e-cigarette products, mostly tobacco and menthol flavored,^7^ with thousands of applications remaining under review.^8,9^ However, the US market remains dominated by non–FDA-authorized flavored e-cigarettes that are particularly popular among youths, with more than 6000 products sold without authorization.^10^

Several states have taken action to regulate non–FDA-authorized e-cigarettes by restricting sales based on PMTA application status. Supported by major tobacco companies, these policies were promoted to reduce illicit products and youth vaping.^11,12,13,14^ As of June 2026, 16 states had enacted laws establishing public, state-managed e-cigarette directories (or registries) (eTable 1 in Supplement 1),^15^ with implementation dates spanning 2022 through 2027 and similar laws proposed in nearly every other state. Alabama (May 2022), Oklahoma (October 2023), and Louisiana (November 2023) were the first to implement directory laws. Louisiana’s directory was suspended during litigation (January 24 to March 17, 2024) before enforcement resumed.^16^ Most directories list products permitted for sale,^15^ while Florida lists prohibited products^17^ and Kentucky lists retailers selling authorized products.^18^

Alabama,^19^ Oklahoma^20^ and Louisiana^21^ permit sales only of products listed in their directories, although submission requirements, product definitions, fees, and enforcement vary. In Alabama and Louisiana, manufacturers must provide one of the following: evidence of an FDA marketing-granted order; a sworn statement that the product was on the market as of August 8, 2016, with a PMTA submitted by September 9, 2020, and proof of FDA receipt; or a sworn statement that a marketing denial appeal is pending. Oklahoma requires only a sworn statement that products received an FDA marketing-granted order or met the 2016-2020 PMTA submission deadlines. E-cigarette definitions also vary. At the time of this study, Alabama’s law covers only tobacco-derived nicotine; Louisiana’s law applies to all e-cigarettes regardless of substance; and Oklahoma’s law does not define e-cigarettes, although other Oklahoma laws apply broadly to all e-cigarettes.^22^ Fee structures also differ: Alabama charges manufacturers a $2000 initial certification fee and $500 annual renewal; Louisiana charges $100 per product annually; and both impose $1000 daily fines on the manufacturer for unlisted product sales. Oklahoma imposes no fees or penalties.

A core challenge for all states is the absence of an independent source to verify manufacturers’ claims. As of June 2026, there was no public list of when products entered the market, and the FDA has not published details on pending PMTAs, marketing denial orders, or appeals.^23^ This lack of verifiable information complicates enforcement and requires reliance on manufacturers’ attestations. Nonetheless, directory inclusion might boost sales by signaling legitimacy or implying FDA authorization.

Retail scanner data provide timely measures of product availability and sales. Although they do not capture online or tobacco specialty stores, they cover a substantial share of the retail marketplace and are widely used in tobacco control research.^24,25,26,27,28,29^ To our knowledge, no study has evaluated the impact of directory laws on e-cigarette sales while accounting for potential confounders. This study used retail scanner data and a synthetic difference-in-differences (SDID) model to evaluate whether directory laws in Alabama, Oklahoma, and Louisiana were associated with changes in e-cigarette nicotine sales and product availability relative to states without such policies. These early-adopter states allow examination of both immediate and evolving effects.

## Methods

### Data Source

This cross-sectional study followed the Strengthening the Reporting of Observational Studies in Epidemiology (STROBE) reporting guideline. The non−human participant data were deemed exempt from oversight by the Advarra Institutional Review Board, and the need for informed consent was waived. The CDC Foundation performed custom research using Circana LLC (Multi-Outlet+ and convenience) retail point-of-sale data from food, drug, mass merchandise, club, dollar, and convenience stores. Circana LLC projects sales from participating retailers to produce state-representative estimates; participating retailers account for approximately 90% of total consumer packaged goods dollar sales based on census point-of-sale data and include most major convenience store chains.^30,31^ However, these data do not capture sales from online retailers or tobacco specialty stores, a common limitation of retail scanner datasets.

The dataset includes weekly unit and dollar sales by universal product code (UPC), along with product attributes (eg, flavor, product type, e-liquid capacity, nicotine strength). Sales were aggregated into 4-week (monthly) periods for the analysis. The initial sample includes 43 states observed over 56 months from the 4-week period ending January 31, 2021, to the 4-week period ending April 20, 2025.

To isolate directory law effects, we excluded 7 states with statewide flavored e-cigarette restrictions.^32^ Florida and Kentucky were also excluded because they enacted directory laws late in the study period, leaving insufficient postpolicy follow-up. States with unenforced directory laws or unpublished directories as of April 2025 (Arkansas, Iowa, Mississippi, Nebraska, North Carolina, Pennsylvania, South Carolina, Tennessee, Virginia, and Wisconsin) remained in the control group, as their policies took effect outside the study window; a sensitivity analysis excluded these states to assess potential contamination. The final sample included 3 intervention and 31 control states, yielding 1904 state-month observations.

### Measures

#### Outcome Variables

The primary outcome was monthly per capita e-cigarette nicotine sales (in milligrams),^33^ calculated as units sold × e-liquid volume (in milliliters) × nicotine concentration (in milligrams per milliliter), summed across UPCs within each state-month and divided by state population. A secondary outcome was product availability, measured as the number of distinct UPCs sold per each state-month. All outcomes were analyzed overall and by flavor (tobacco vs nontobacco) and product type (disposables vs prefilled cartridges). Because directory-listed products were primarily prefilled cartridges and largely nontobacco flavored (see Results section), these stratifications helped explain the overall effects and assess potential substitutions.

#### Policy Exposure

An indicator identified each intervention state after directory publication. These dates included May 2022 for Alabama, October 2023 for Oklahoma, and November 2023 for Louisiana.

### Covariates

Louisiana tripled its e-cigarette excise tax from $0.05/mL to $0.15/mL effective July 1, 2023, 4 months before directory publication.^34^ Neither Alabama nor Oklahoma taxed e-cigarettes during the study period. We controlled for standardized e-cigarette taxes per milliliter for closed-system devices (disposables and prefilled cartridges), following the method of Cotti et al,^35^ and updated tax rates through April 2025 using various sources.^36,37,38,39^ Ad valorem taxes were converted to per-milliliter rates, and monthly tax measures accounted for midmonth rate changes by weighting each rate by the share of the month it was in effect. Results were robust to using open-system tax rates. Additional covariates included unemployment rates,^40^ tobacco control funding,^41^ smoking cessation coverage,^41^ smoke-free laws,^41^ COVID-19 cases, and state and year-month fixed effects.

### Statistical Analysis

This study used the SDID method to estimate the average treatment effect of e-cigarette directory laws on treated states (ATT). SDID optimally weights states and time periods to create a synthetic control that matches pretreatment trends.^42,43^ Unlike standard DID, SDID requires parallel trends only between treated states and their synthetic controls. Unlike the synthetic control method, SDID allows differences in preintervention levels, requiring only trend alignment.^42,43^ SDID has been shown to reduce bias and improve precision and is increasingly used in tobacco policy evaluations.^27,28,29^

To assess effects over time, we estimated month-specific effects using event-study SDID regressions,^44^ comparing outcome differences between treated states and SDID-generated synthetic controls in each month relative to a weighted mean of pretreatment differences. Preintervention coefficients serve as a placebo test; nonsignificant values supported parallel trends.

We conducted separate SDID analyses for Alabama, Oklahoma, and Louisiana to estimate the associations of directory laws with per-capita e-cigarette nicotine sales and the number of distinct UPCs (both log-transformed), overall and by product and flavor type, adjusting for the covariates described above. ATTs were reported as percentage changes using (exp [SDID coefficient] − 1) × 100. Statistical inference used placebo-based permutation tests, comparing observed effects with a null distribution generated by sequentially assigning placebo treatments to control states.^42^ SEs and *P* values were based on 1000 permutations. Statistical significance was set at 2-sided *P* < .05 for all analyses. Analyses were conducted from August 1, 2025, to July 20, 2026, using Stata, version 17 (StataCorp LLC).

## Results

### Descriptive Trends

**Figure 1.**
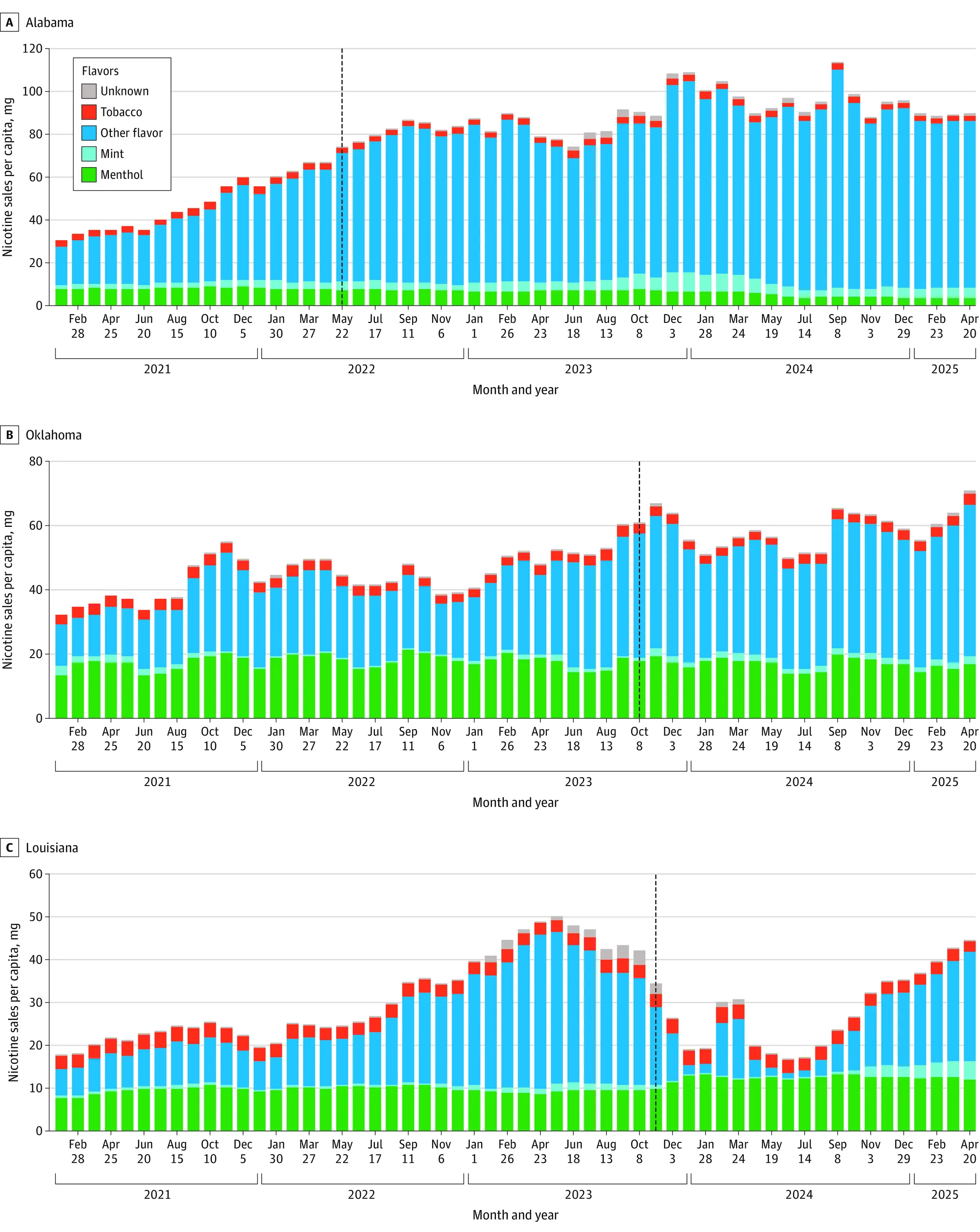
Bar Graphs Showing E-Cigarette Nicotine Sales by Flavor Per capita e-cigarette nicotine sales were calculated as units sold × e-liquid volume (in milliliters) × nicotine concentration (in milligrams per milliliter), summed across products per state-month and divided by state population. Each bar represents a 4-week aggregate interval. Vertical dashed lines indicate the date each directory was published.

**Figure 2.**
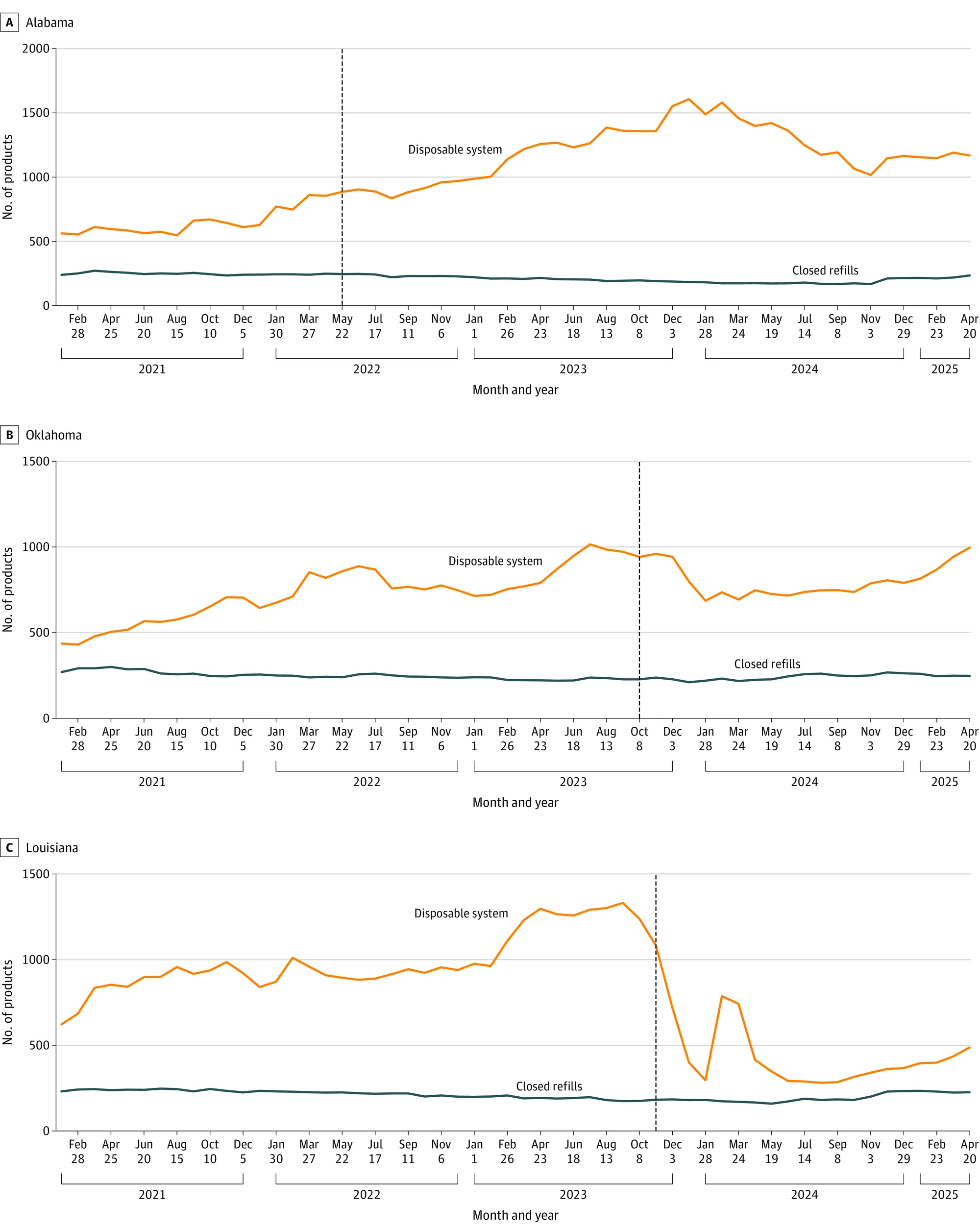
Line Graphs Showing Number of Distinct E-Cigarette Products on the Market by Product Type Measured as the number of distinct universal product codes sold during each state-month. Each date in the x-axis represents a 4-week aggregate interval. Vertical dashed lines indicate the date each directory was published.

A total of 1904 state-observations were included in the analysis. Trends in e-cigarette nicotine sales and product availability were broadly similar in Alabama and Oklahoma but differed in Louisiana. In Alabama, per-capita nicotine sales rose after the May 2022 directory publication from 47.8 mg (January 2021 to April 2022) to 89.6 mg (May 2022 to April 2025) (Figure 1A). Nontobacco flavors consistently exceeded 90%, reaching 96.1% by April 2025. The share of disposable sales grew from 75.6% to 90.2% (eFigure 1 in Supplement 1), and product availability expanded from 910 to 1414 products, driven by flavored disposables (Figure 2A).

In Oklahoma, per-capita nicotine sales fluctuated after the October 2023 directory but increased overall from 45.0 mg (January 2021 to September 2023) to 59.3 mg (October 2023 to April 2025) (Figure 1B). Nontobacco flavors remained at greater than 90%, reaching 93.6% by April 2025. Disposables increased from 53.0% to 66.4% (eFigure 1 in Supplement 1), and product availability grew from 981 to 1059 products, driven by flavored disposables (Figure 2B).

In Louisiana, sales and product availability shifted substantially. After the July 2023 tax increase, per-capita nicotine sales fell to 42.1 mg in October 2023 (from 48.2 mg in June 2023) (Figure 1C), while product availability remained stable at 1421 products (Figure 2C). After the November 2023 directory publication, per-capita nicotine sales decreased to their lowest level of 16.9 mg by June 2024, driven by flavored disposables (eFigure 1 in Supplement 1). Meanwhile, prefilled cartridge sales, especially menthol, surged, with cartridge share rising from 29.5% to 91.6% and menthol share from 22.4% to 73.2%. Product availability fell to 474 products. From June 2024 to April 2025, per-capita nicotine sales rebounded to 44.9 mg, surpassing predirectory levels. Although menthol and prefilled cartridge sales remained relatively stable, their shares fell to 27.1% and 33.0%, respectively, as total sales grew. Product availability increased to 742 products, led by flavored disposables. Nontobacco flavors stayed dominant (>80%), reaching 93.2% by April 2025.

### SDID Effects

**Figure 3.**
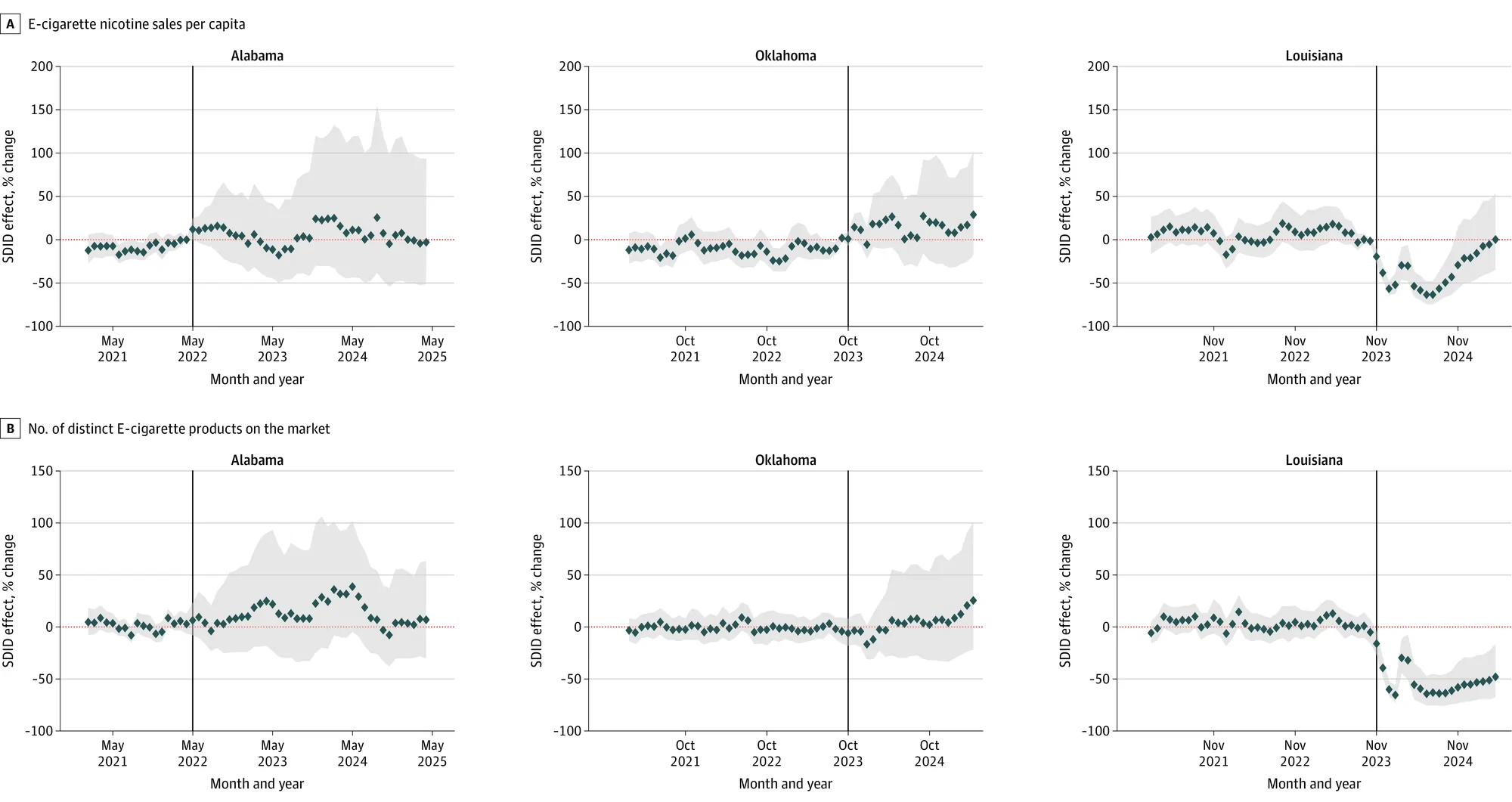
Scatterplots Showing Change Over Time in the Association of Directory Laws With E-Cigarette Nicotine Sales and Number of Distinct Products Sold Data were analyzed as a synthetic difference-in-differences (SDID) event study. Vertical lines denote directory law implementation dates. Shaded areas represent 95% CIs.

eTable 2 in Supplement 1 lists the top donor states and weights used to construct synthetic controls. Trends between treated states and synthetic controls were similar prior to publication, supporting the parallel trends assumption (Figure 3 and eFigure 2 in Supplement 1).

In Alabama, postdirectory trends for sales and product availability closely matched their synthetic controls (eFigure 2 in Supplement 1), yielding no associations overall or stratified by product and flavor type (Table). Similarly, no associations were observed in Oklahoma for overall sales or product availability; however, sales of menthol-flavored prefilled cartridges increased relative to the synthetic control (ATT, 21.74% [95% CI, 5.55% to 40.42%]). Event study analyses showed that the null overall associations with sales and product availability in both states persisted through April 2025 (Figure 3).

**Table.** Association of Directory Laws With State Monthly E-Cigarette Nicotine Sales and the Number of Distinct Products on the Market by Flavor and Product Type—SDID Average Treatment Effects^a^

	Alabama		Oklahoma		Louisiana	
	ATT (95% CI), %	*P* value	ATT (95% CI), %	*P* value	ATT (95% CI), %	*P* value
**E-cigarette nicotine sales per capita** ^b^						
Overall	5.83 (−34.62 to 71.31)	.82	13.58 (−14.48 to 50.86)	.38	−38.29 (−53.10 to −18.80)	<.001
By flavor						
Tobacco	−3.58 (−21.92,19.06)	.73	0.11 (−13.28 to 15.57)	.99	9.36 (−7.94 to 29.92)	.31
Nontobacco	1.43 (−40.75 to 73.64)	.96	15.98 (−17.36 to 62.75)	.39	−37.39 (−54.06 to −14.66)	<.001
By product						
Disposables	−23.58 (−79.71 to 187.88)	.69	7.92 (−54.98 to 158.69)	.86	−74.11 (−88.75 to −40.44)	<.001
Prefilled cartridges	−12.80 (−29.53 to 7.90)	.21	0.80 (−18.06 to 24.00)	.94	41.45 (13.72 to 75.93)	<.001
By flavor and product						
Tobacco disposables	78.70 (−27.94 to 343.14)	.21	25.08 (−49.98 to 212.81)	.63	−25.43 (−72.44 to 101.77)	.56
Nontobacco disposables	−27.31 (−82.51 to 202.13)	.66	7.87 (−55.95 to 164.13)	.87	−71.55 (−87.84 to −33.48)	<.001
Tobacco prefilled cartridges	−3.43 (−20.90 to 17.89)	.73	1.21 (−11.54 to 15.80)	.86	11.94 (−2.68 to 28.75)	.11
Menthol prefilled cartridges	−12.77 (−32.95 to 13.48)	.31	5.91 (−18.73 to 38.02)	.67	50.24 (15.25 to 95.84)	<.001
**No. of distinct E-cigarette products** ^c^						
Overall	13.48 (−15.15 to 51.76)	.39	1.50 (−23.98 to 35.53)	.92	−53.55 (−65.67 to −37.14)	<.001
By flavor						
Tobacco	−7.41(−17.44 to 3.84)	.19	−1.85 (−14.72 to 12.97)	.80	−0.72 (−14.55 to 15.35)	.93
Nontobacco	12.94 (−20.97 to 61.41)	.50	4.04 (−27.14 to 48.56)	.83	−61.02 (−73.30 to −43.10)	<.001
By product						
Disposables	10.51 (−30.58 to 75.92)	.67	−4.41 (−38.45 to 48.45)	.84	−63.45 (−76.81 to −42.42)	<.001
Prefilled cartridges	−7.11 (−19.39 to 7.05)	.31	14.91 (2.53 to 28.79)	.02	4.25 (−7.48 to 17.46)	.49
By flavor and product						
Tobacco disposables	−24.49 (−49.75 to 13.45)	.24	−35.93 (−66.58 to 22.82)	.18	−26.46 (−59.34 to 33.01	.31
Nontobacco disposables	11.66 (−30.67 to 79.85)	.73	−4.77 (−40.27 to 51.83)	.84	−64.19 (−77.97 to −41.80)	<.001
Tobacco prefilled cartridges	−4.77 (−18.32 to 11.02)	.53	10.18 (−1.51 to 23.26)	.09	9.00 (−3.22 to 22.77)	.16
Menthol prefilled cartridges	−7.88 (−22.83 to 9.97)	.36	21.74 (5.55 to 40.42)	.01	−0.28 (−14.72 to 16.60)	.97

Abbreviations: ATT, average treatment effect of directory publication; SDID, synthetic difference-in-differences.

^a^ Associations are represented by the ATT, which was estimated using SDID regression for each treated state (Alabama, Oklahoma, and Louisiana) compared with its synthetic control. Outcomes were log-transformed; ATTs were expressed as percentage of change, calculated as (exp [SDID coefficient] − 1) × 100. Positive values indicate percentage increases due to directory publication; negative values indicate declines. The full model included state and year-month fixed effects, e-cigarette tax per milliliter, unemployment rates, tobacco control funding, state coverage of smoking cessation services, smoke-free laws, and COVID-19 cases. E-cigarette sales data were obtained from Circana LLC, point-of-sale retail scanner data (Multi-Outlet+ and convenience); data for internet and vape shop sales were not available. E-cigarette directories (state-managed lists of registered products permitted for sale) were published in Alabama in May 2022, in Oklahoma in October 2023, and in Louisiana in November 2023.

^b^ Calculated as units sold × e-liquid volume (in milliliters) × nicotine concentration (in milligrams per milliliter), summed monthly by state, and divided by state population.

^c^ Calculated as the number of unique Universal Product Codes per month per state.

In Louisiana, the directory law was associated with a reduction in per capita nicotine sales relative to the synthetic control (ATT, −38.29% [95% CI, −53.10% to −18.80%]). Event-study estimates indicated that this association was temporary. Relative to the synthetic control, per capita nicotine sales were reduced by 19.46% (95% CI, −27.32% to −10.76%) immediately after directory publication, reached a maximum reduction of 63.43% (95% CI, −74.32% to −47.92%) in July 2024, and then steadily rebounded. By October 2024, sales were reduced by 43.01% (95% CI, −63.48% to −11.07%) and became nonsignificantly different thereafter. By April 2025, sales increased above predirectory levels by 0.23% (95% CI, −34.53% to 53.45%) relative to the synthetic control. In contrast, product availability declined consistently. On average, directory publication was associated with an ATT reduction in product availability of 53.55% (95% CI, −65.67% to −37.14%). Event-study estimates indicated that the number of products sold was 63.07% lower than the synthetic control in July 2024 (95% CI, −75.22% to −44.98%) and remained 61.23% lower in October 2024 (95% CI, −74.23% to −41.67%). Although the difference decreased to 47.89% by April 2025 (95% CI, −67.49% to −16.49%), it remained statistically significant.

Stratified analyses in Louisiana revealed contrasting patterns. Sales and availability of nontobacco-flavored disposable products declined (ATT, −71.55% [95% CI, −87.84% to −33.48%] and −64.19% [95% CI, −77.97% to −41.80%] on average, respectively), whereas sales of menthol prefilled cartridge increased by 50.24% (95% CI, 15.25% to 95.84%), with no change in availability. This increase was driven largely by a specific menthol vaping brand (Vuse Alto; R. J. Reynolds Vapor Co) that lacks FDA marketing authorization.

### Sensitivity Analyses

Sensitivity analyses confirmed the robustness of the estimates (eTable 3 in Supplement 1). Results were consistent across model specifications, regardless of whether covariates were included, after excluding control states with unenforced directory laws, excluding Louisiana’s suspension period, using alternative numbers of permutation iterations (1000, 5000, and 10 000), using an alternative sales measure (milliliters of e-liquid), and applying alternative estimation methods (DID and synthetic controls).

While DID and synthetic controls produced negative estimates comparable to SDID, neither detected an association of Louisiana’s directory law with e-cigarette nicotine sales. Estimated reductions ranged from an ATT of −29.01% (95% CI, −57.56% to 18.76%; DID) to −48.90% (95% CI, −75.52% to 6.66%; synthetic controls), with an ATT at −38.29% (95% CI, −53.10% to −18.80%) for SDID. However, the 95% CIs for DID and synthetic controls were substantially wider than those of SDID, suggesting greater precision from SDID’s improved pretreatment balance through unit and time weighting.

### Listed vs Unlisted Products

To assess market dynamics and potential enforcement patterns, we classified products as listed or unlisted based on state directories accessed in July 2026 because information on the dates of initial directory inclusion was unavailable or incomplete.^45,46,47^ As of April 2025, unlisted products accounted for 92.1% of per capita e-cigarette nicotine sales in Alabama, 53.6% in Oklahoma, and 52.3% in Louisiana (eTable 4 in Supplement 1). Listed product sales were largely prefilled cartridges in nontobacco flavors, particularly menthol.

**Figure 4.**
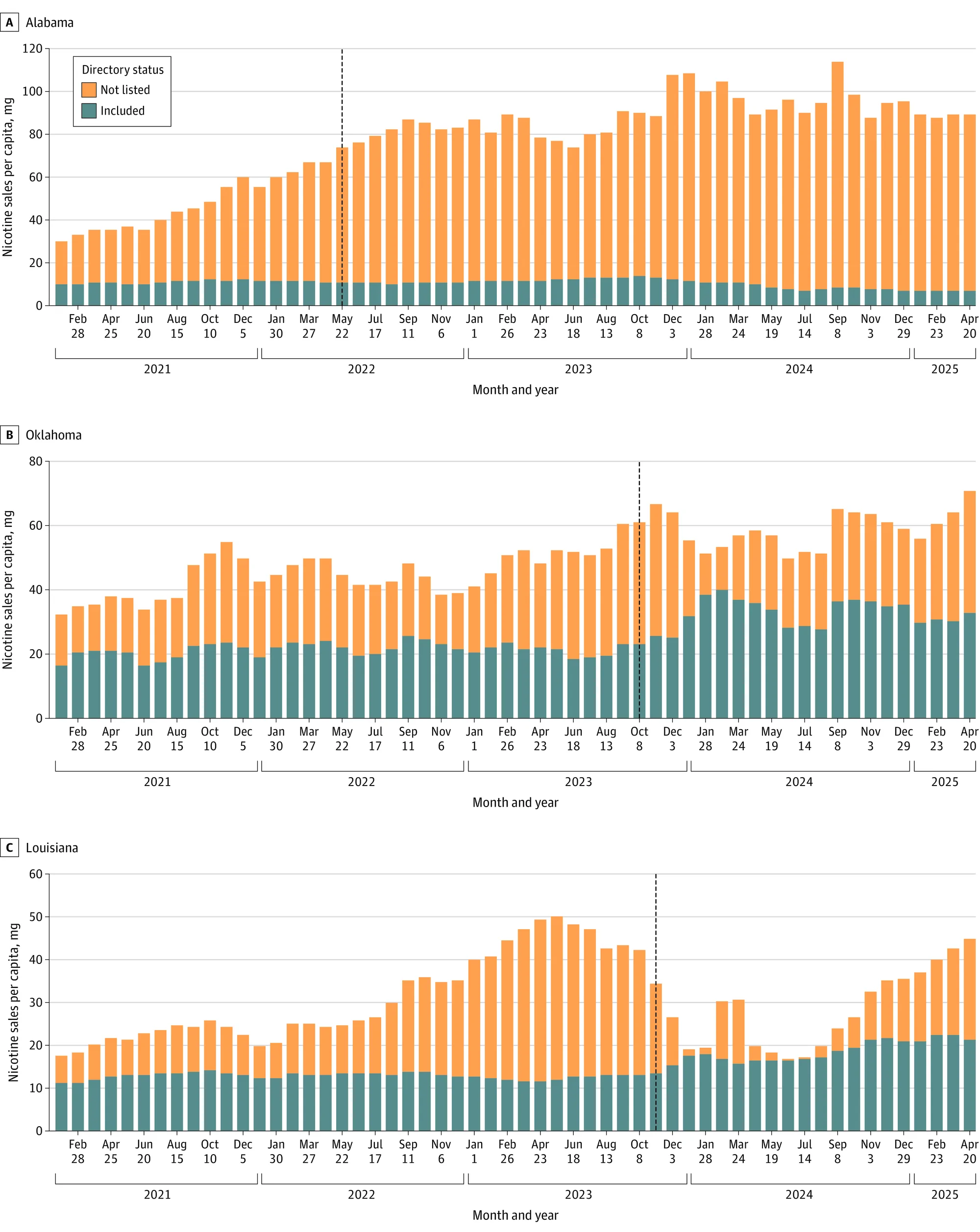
Bar Graphs Showing Trends in State Monthly E-Cigarette Nicotine Sales by Directory Status Products were classified as listed or unlisted based on published state directories as of July 19, 2026. Each bar represents a 4-week aggregate interval. Vertical dashed lines indicate the date each directory was published.

Trends in unlisted product sales varied across states. In Alabama, unlisted product sales remained dominant and continued to increase following directory publication. In Oklahoma, unlisted sales remained stable for 2 months after directory publication, then declined while still accounting for about one-quarter of per capita sales before rebounding to predirectory levels. In contrast, Louisiana experienced a sharp decline in unlisted product sales immediately following directory publication. Unlisted sales remained low for several months before gradually rebounding, suggesting that enforcement may have weakened over time (Figure 4).

## Discussion

This study is the first, to our knowledge, to examine changes in e-cigarette nicotine sales following the implementation of e-cigarette directory laws and found no sustained changes associated with these laws. Among the 3 early-adopting states, only Louisiana experienced an initial decline in nicotine sales, which later reversed. By April 2025, more than half of sales in all 3 states were for products not listed in directories. Overall, these directory laws do not appear to sustainably reduce sales of flavored e-cigarettes, which are particularly appealing to youths and young adults.

In Oklahoma and Alabama, no associations between directory publication and e-cigarette sales or product availability were detected; flavored disposable products continued to dominate the market. In Louisiana, the law was associated with a sustained decline in product availability, but this did not translate into sustained reductions in sales. Disposable product sales initially declined, while menthol-flavored prefilled cartridge sales—driven primarily by the Vuse Alto brand—increased and partially offset those declines. By April 2025, menthol cartridge sales remained elevated, and flavored disposable sales had rebounded.

Little public information is available on these states’ enforcement practices, and none are required to issue annual implementation or enforcement reports.^19,20,21^ Louisiana may have experienced an initial impact, unlike Alabama and Oklahoma, because it requires manufacturers to pay a fee to register each product rather than a flat fee and imposes substantial fines for selling unlisted products. However, sales reductions were not sustained, despite no apparent changes to these provisions. It remains unclear whether Louisiana’s initial decline resulted from outreach, enforcement efforts, or other factors, including shifts in sales to retailers not captured in the data. Further research, including qualitative and administrative data and information on untracked sales channels, is needed to better understand how implementation and enforcement shape market responses to directory laws.

Louisiana’s reduction in product availability also warrants cautious interpretation. A reduction in the number of available products does not necessarily translate into improved public health outcomes; the youth vaping epidemic began during a period when relatively few e-cigarette products were available. Unless accompanied by sustained reductions in sales, reductions in product availability may primarily benefit major tobacco companies by reducing competition.

Our findings suggest that directory laws may perpetuate, rather than eliminate, the sale of youth-appealing products not authorized by the FDA. As a strategy supported by the tobacco industry,^11,12,13,14^ their lack of sustained impact on sales aligns with extensive literature showing that industry-backed strategies are ineffective at reducing tobacco use across the lifespan.^48,49,50,51^ Directory laws also impose significant administrative and financial burdens on states; 2025 legislative analyses estimated implementation and enforcement costs exceeding $8 million in Oklahoma^52^ and $27 million for a proposed Texas bill.^53^ These laws also create compliance challenges for retailers, who must track changing product lists that differ from the FDA’s authorized list.^19,20,21^

Directory laws may further impose opportunity costs by diverting limited state resources from evidence-based tobacco control efforts. They may also give policymakers and the public the false impression that tobacco-related concerns have been resolved. Proven interventions, including comprehensive restrictions on flavored tobacco product sales,^24,25,26,54^ comprehensive smoke-free policies, and substantial price increases, remain more effective at reducing tobacco sales and use and preventing youth initiation.^48,49^ States can address unauthorized sales by prohibiting online sales, requiring licensure across the distribution chain, and sharing information about potential violations with the FDA.

### Limitations

This study has limitations. First, scanner data do not capture e-cigarette sales from vape shops or online retailers, where trends may differ. However, these channels were found to sell unauthorized products and violate sales restrictions more than mainstream retailers,^55,56,57^ which are better represented in scanner data. Therefore, our finding of no sustained impact of directory laws on e-cigarette sales is likely conservative unless these laws substantially affected these less compliant, untracked channels. Second, labeled nicotine content may not reflect actual levels; however, results were consistent across alternative sales measures. Third, unmeasured factors may have influenced the results, although the SDID approach helped reduce bias and yield consistent findings across sensitivity analyses. Fourth, estimates relied on data that were available during our analyses, and subsequent updates may differ. Fifth, product classification may have overestimated listed-product sales because some products appearing on state directories accessed in July 2026 may not have been listed during the study period. In addition, coding was conducted at the brand rather than the product level, which may have further overestimated listed-product sales if only some products within a brand were directory eligible. Consequently, estimates of unlisted product sales are conservative. Despite this limitation, unlisted products accounted for most e-cigarette sales by April 2025 in all 3 study states. Finally, limited information on enforcement practices makes it difficult to assess their effect on sales.

## Conclusions

In this cross-sectional study, directory laws were not associated with a sustainable reduction of the sale of illegally marketed e-cigarettes, including flavored products appealing to youths and young adults. Instead, directory laws might divert attention from evidence-based tobacco control measures known to reduce tobacco use, such as price increases, smoke-free air laws, and comprehensive restrictions on flavored product sales.

## Notes

Supplement 1. **eFigure 1.** E-Cigarette Nicotine Sales by Product and Flavor **eFigure 2.** Trends in State Monthly E-Cigarette Nicotine Sales and Number of Distinct Products on the Market: Treated States vs Synthetic Controls Before and After Directory Publication **eTable 1.** State E-Cigarette Directory Law Implementation Dates and Enforcement Status as of June 2026 **eTable 2.** Synthetic Difference-in-Differences (SDID) Unit Weights: Top-Contributing Donor States and Corresponding Weights **eTable 3.** The Effect of Directory Laws on E-Cigarette Nicotine Sales and the Number of Distinct Products on the Market: Sensitivity Analysis **eTable 4.** Overview of State Directory Listings and E-Cigarette Nicotine Sales: April 2025

Supplement 2.
Data Sharing Statement
