# Supplementary material for: E-Cigarette Directory Laws, Sales, and Product Availability From 3 Early-Adopting US States
Source: JAMA Netw Open. 2026 Sep 15;9(9):e2633874. doi: 10.1001/jamanetworkopen.2026.33874 (PMC13579438; doi:10.1001/jamanetworkopen.2026.33874)
Supplement: Supplementary file 1 [file jamanetwopen-e2633874-s001.pdf]

Supplementary Online Content

Ali FRM, Mahoney M, Jones ELS, Tynan MA, Marynak K. E-cigarette directory laws, sales, and product availability from 3 early-adopting US states. *JAMA Netw Open.* 2026;9(9):e2633874. doi:10.1001/jamanetworkopen.2026.33874

eFigure 1. E-Cigarette Nicotine Sales by Product and Flavor
eFigure 2. Trends in State Monthly E-Cigarette Nicotine Sales and Number of Distinct Products on the Market: Treated States vs Synthetic Controls Before and After Directory Publication
eTable 1. State E-Cigarette Directory Law Implementation Dates and Enforcement Status as of June 2026
e**Table 2.** Synthetic Difference-in-Differences (SDID) Unit Weights: Top-Contributing Donor States and Corresponding Weights
eTable 3. The Effect of Directory Laws on E-Cigarette Nicotine Sales and the Number of Distinct Products on the Market: Sensitivity Analyses
eTable 4. Overview of State Directory Listings and E-Cigarette Nicotine Sales: April 2025

This supplementary material has been provided by the authors to give readers additional information about their work.

© 2026 Ali FRM et al. *JAMA Network Open*.

eFigure 1. E-cigarette Nicotine Sales by Product and Flavor

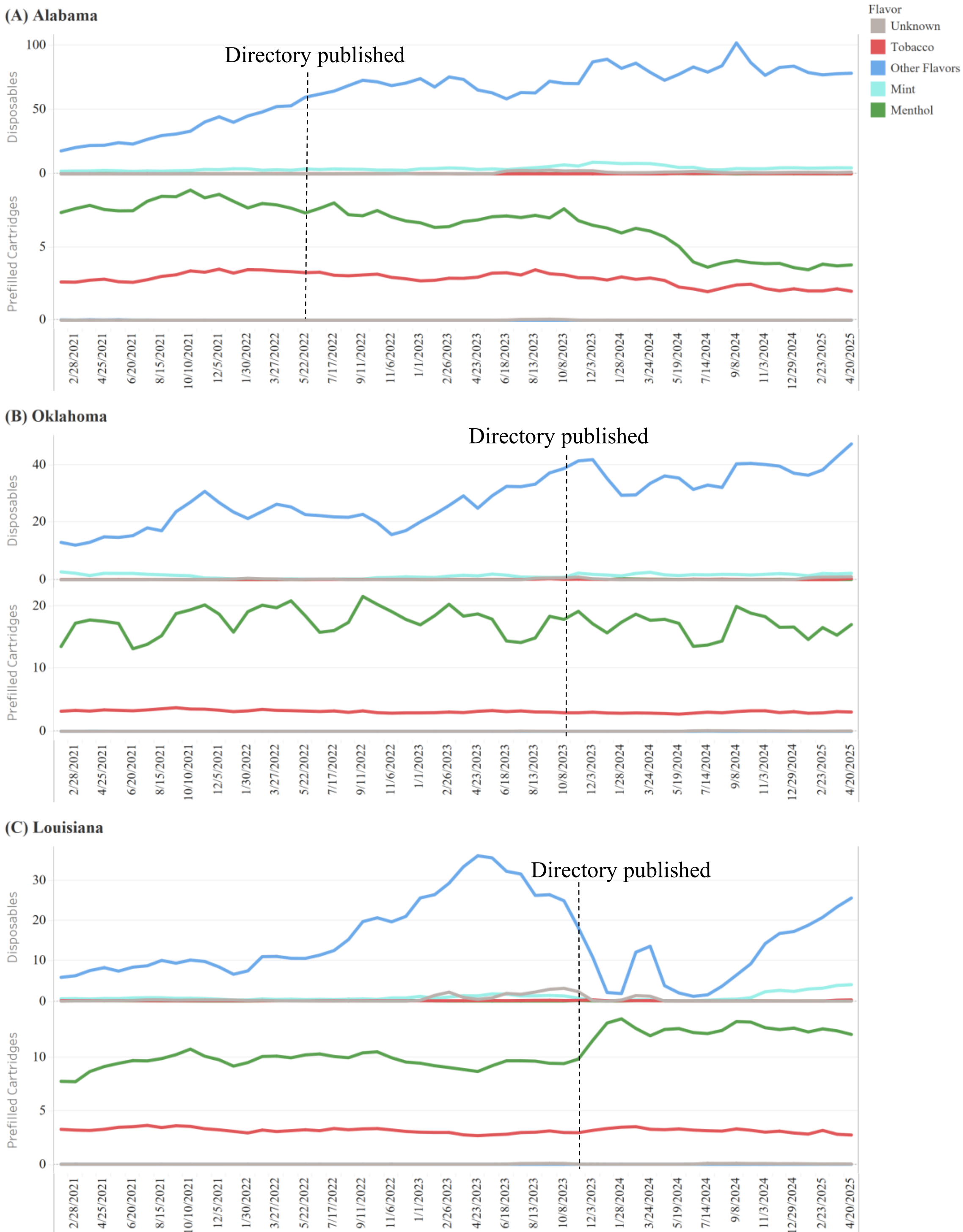

Note: E-cigarette sales data were obtained from Circana, LLC, point-of-sale (POS) retail scanner data (Multi-Outlet+ and Convenience); Internet and vape shop sales were not available. Prefilled cartridges include tanks, cartridges and pods for rechargeable/reusable devices; disposables are non-rechargeable, non-refillable devices. E-cigarette nicotine sales (mg per capita) were calculated as: units sold × e-liquid volume (mL) × nicotine concentration (mg/mL), summed monthly by state and divided by state population. Each tick represents a 4-week aggregate interval. E-cigarette directories—state-managed lists of registered products permitted for sale—were published in AL (May 2022), OK (Oct 2023) and LA (Nov 2023).

© 2026 Ali FRM et al. *JAMA Network Open*.

eFigure 2. Trends in State Monthly E-Cigarette Nicotine Sales and Number of Distinct Products on the Market: Treated States vs Synthetic Controls Before and After Directory Publication

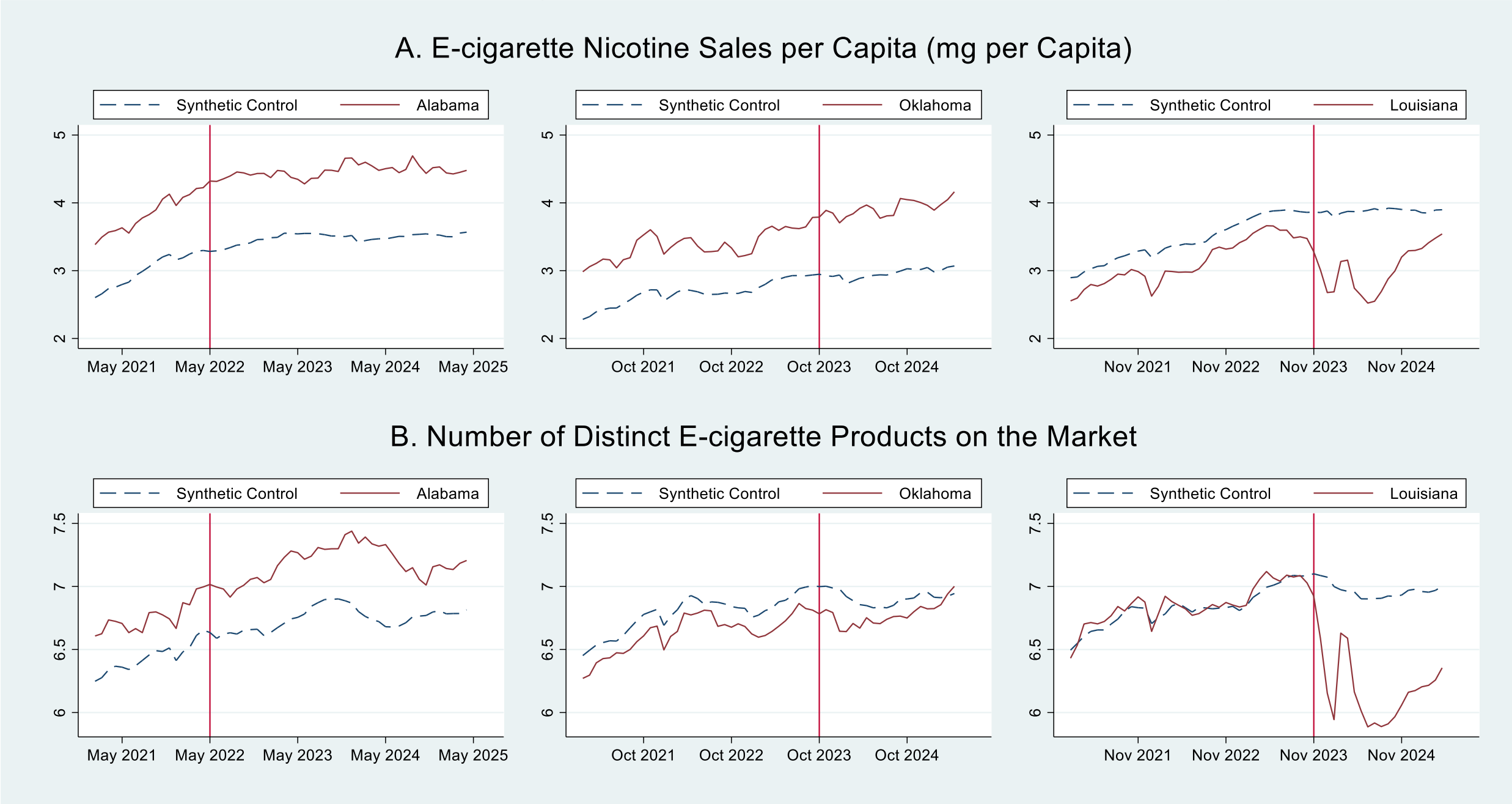

Note: E-cigarette sales data were obtained from Circana, LLC, point-of-sale (POS) retail scanner data (Multi-Outlet+ and Convenience); Internet and vape shop sales were not available. E-cigarette directories—state-managed lists of registered products permitted for sale—were published in AL (May 2022), OK (Oct 2023) and LA (Nov 2023). Vertical solid lines mark the month each directory was published. Outcomes were: (1) e-cigarette nicotine sales (milligram per capita) and (2) number of distinct products (unique UPCs per month per state). Nicotine sales were calculated as units sold × e-liquid volume (mL) × nicotine concentration (mg/mL), summed monthly by state, and divided by state population. Both outcomes were log-transformed. The Y-axis displays adjusted log values for treated states (AL, OK, LA) and their synthetic controls, estimated using synthetic difference-in-differences (SDID) models with state and year-month fixed effects, e-cigarette tax per milliliter, unemployment rates, tobacco control funding, state coverage of smoking cessation services, smoke-free laws, and COVID-19 cases

© 2026 Ali FRM et al. *JAMA Network Open*.

eTable 1. State E-Cigarette Directory Law Implementation Dates and Enforcement Status as of June 2026

State	Directory Effective/Published	Notes
Alabama	May 2022	Directory implemented.
Oklahoma	October 2023	Directory implemented.
Louisiana	November 2023	Directory implemented; enforcement temporarily suspended during litigation (January 24–March 17, 2024) before resuming.
Kentucky	January 2025	Directory implemented; state directory lists retailers claiming to sell authorized products.
Utah	January 2025	Enforcement delayed by litigation until March 24, 2025.
Florida	March 2025	Directory implemented; state directory lists prohibited rather than authorized products.
North Carolina	May 2025	Directory implemented.
Wisconsin	September 2025	Directory implemented.
Mississippi	October 2025	Directory implemented.
Nebraska	October 2025	Directory implemented.
Arkansas	November 2025	Directory implemented.
Virginia	January 2026	Directory implemented.
Pennsylvania	October 2026	Directory scheduled for implementation.
Tennessee	January 2027	Directory scheduled for implementation.
South Carolina	February 2027	Directory scheduled for implementation.
Iowa	—	Directory law enacted but not enforced due to litigation.

Note: Authors' compilation based on information from the Public Health Law Center's State E-Cigarette Registry Bill Map (accessed June 15, 2026). https://www.publichealthlawcenter.org/resources/state-e-cigarette-registry-bill-map. Most state directories list products authorized for sale. Florida's directory lists prohibited products, while Kentucky's directory lists retailers claiming to sell authorized products. Iowa enacted a directory law, but enforcement has not commenced because of ongoing litigation. Utah's enforcement was delayed until March 24, 2025, and Louisiana's directory was temporarily suspended during litigation from January 24 to March 17, 2024.

© 2026 Ali FRM et al. *JAMA Network Open*.

eTable 2. Synthetic Difference-in-Differences (SDID) Unit Weights: Top-Contributing Donor States and Corresponding Weights[a]

Alabama[b]				Oklahoma[b]				Louisiana[b]			
E-cigarette Nicotine Sales[c]		Product Availability[d]		Nicotine Sales		Product Availability		Nicotine Sales		Product Availability	
State	%	State	%	State	%	State	%	State	%	State	%
South Dakota	16.5	Iowa	10.9	Nevada	26.3	Virginia	10.8	Oregon	32.7	Nebraska	11.9
South Carolina	14.5	Mississippi	9.3	New Mexico	12.1	New Mexico	9.3	Arizona	8.8	Arizona	11.1
Michigan	14.4	Missouri	9.1	Pennsylvania	11.7	Michigan	9.0	Mississippi	8.6	Georgia	9.9
Arkansas	11.2	New Mexico	8.9	West Virginia	9.1	New Hampshire	8.2	Arkansas	7.9	Vermont	9.1
Oregon	9.2	Nevada	7.7	Arizona	6.8	North Carolina	5.8	Pennsylvania	7.7	Kansas	6.7
Tennessee	8.1	West Virginia	6.2	South Dakota	6.1	Illinois	5.6	Minnesota	6.4	Virginia	6.6
Kansas	5.6	Indiana	6.0	Ohio	5.9	Missouri	5.2	Illinois	6.3	Minnesota	6.5
New Mexico	4.7	Kansas	4.5	Kansas	5.3	Nebraska	5.1	West Virginia	5.7	Connecticut	6.1
North Carolina	4.0	New Hampshire	3.8	Michigan	4.0	Arkansas	5.0	Wyoming	4.7	Nevada	5.3
Georgia	3.9	Tennessee	3.8	Indiana	3.1	Indiana	5.0	Vermont	4.5	Oregon	5.3
Vermont	2.7	Maine	3.8	Virginia	2.8	Maine	4.9	Colorado	4.1	Texas	4.8
Iowa	2.6	South Dakota	3.6	Wyoming	2.6	Nevada	4.7	Indiana	2.0	Michigan	3.7
Minnesota	2.2	Arizona	3.4	Nebraska	2.2	Ohio	3.9	Connecticut	0.4	South Dakota	3.4
Missouri	0.2	Vermont	3.4	Tennessee	0.9	Connecticut	3.6	New Hampshire	0.1	Illinois	2.5
Wisconsin	0.1	Virginia	3.1	Illinois	0.9	Wyoming	3.5			Wyoming	2.4

Note: E-cigarette sales data were obtained from Circana, LLC, point-of-sale (POS) retail scanner data (Multi-Outlet+ and Convenience); Internet and vape shop sales were not available.

[a] In synthetic difference-in-differences (SDID) regression, each treated state (AL, OK, LA) was compared to its synthetic control. Full model included state and year-month fixed effects, e-cigarette tax per milliliter, unemployment rates, tobacco control funding, state coverage of smoking cessation services, smoke-free laws, and COVID-19 cases.

[b] These represent the first three states to enact and publish e-cigarette directories—state-managed lists of registered products permitted for sale; AL (May 2022), OK (Oct 2023) and LA (Nov 2023).

[c] E-cigarette nicotine sales were calculated as units sold × e-liquid volume (mL) × nicotine concentration (mg/mL), summed monthly by state, and divided by state population.

[d] Number of distinct products (UPCs) on the market per month per state.

© 2026 Ali FRM et al. *JAMA Network Open*.

eTable 3. The Effect of Directory Laws on E-Cigarette Nicotine Sales and the Number of Distinct Products on the Market: Sensitivity Analyses[a,b,c]

	Alabama		Oklahoma		Louisiana	
	ATT[e] (% Change)	P-value	ATT (% Change)	P-value	ATT (% Change)	P-value
**1) Adjustment for Covariates[d]**						
- **E-cigarette Nicotine Sales per Month (mg per Capita)**						
o Covariates included (Main Model)	5.83	0.82	13.58	0.38	-38.29	<0.001
o Covariates excluded	6.65	0.78	1.79	0.91	-44.47	<0.001
- **Number of Distinct E-cigarette Products on the Market**						
o Covariates included (Main Model)	13.48	0.39	1.50	0.92	-53.55	<0.001
o Covariates excluded	18.31	0.29	-5.93	0.68	-55.41	<0.001
**2) Excluding States Whose Directory Laws Took Effect Outside the Study Period[f]**						
- **E-cigarette Nicotine Sales per Month (mg per Capita)**						
o States included in the control group (Main Model)	5.83	0.82	13.58	0.38	-38.29	<0.001
o States excluded from the control group	7.23	0.80	16.80	0.34	-38.30	<0.001
- **Number of Distinct E-cigarette Products on the Market**						
o States included in the control group (Main Model)	13.48	0.39	1.50	0.92	-53.55	<0.001
o States excluded from the control group	14.72	0.37	-3.89	0.83	-54.83	<0.001

© 2026 Ali FRM et al. *JAMA Network Open*.

**3) Alternative Permutation Iterations (p-value)**						
- **E-cigarette Nicotine Sales per Month (mg per Capita)**						
o 1,000 iterations (Main Model)	5.83	0.82	13.58	0.38	-38.29	<0.001
o 5,000 iterations	5.83	0.82	13.58	0.39	-38.29	<0.001
o 10,000 iterations	5.83	0.82	13.58	0.39	-38.29	<0.001
- **Number of Distinct E-cigarette Products on the Market**						
o 1,000 iterations (Main Model)	13.48	0.39	1.50	0.92	-53.55	<0.001
o 5,000 iterations	13.48	0.39	1.50	0.92	-53.55	<0.001
o 10,000 iterations	13.48	0.39	1.50	0.92	-53.55	<0.001
**4) Alternative E-cigarette Sales Measures**						
- **Nicotine Sales (mg per Capita (Main Model)**	5.83	0.82	13.58	0.38	-38.29	<0.001
- **E-liquid Sales (mL per Capita)[g]**	2.14	0.93	12.86	0.38	-36.75	<0.001
**5) Excluding the two-month suspension period from the Louisiana regression**						
- **E-cigarette Nicotine Sales per Month (mg per Capita)**						
o Include the suspension period (Main Model)	n.a	n.a	n.a	n.a	-38.29	<0.001
o **Exclude the suspension period**	n.a	n.a	n.a	n.a	-39.62	<0.001
- **Number of Distinct E-cigarette Products on the Market**						
o **Include the suspension period (Main Model)**	n.a	n.a	n.a	n.a	-53.55	<0.001
o Exclude the suspension period	n.a	n.a	n.a	n.a	-55.34	<0.001
**6) Alternative Regression Methods**						

© 2026 Ali FRM et al. *JAMA Network Open*.

- **E-cigarette Nicotine Sales per Month (mg per Capita)**						
○ SDID (Main Model)	5.83	0.82	13.58	0.38	-38.29	<0.001
○ DID	31.08	0.34	34.16	0.26	-29.01	0.19
○ SC	22.21	0.66	43.40	0.35	-48.90	0.07
- **Number of Distinct E-cigarette Products on the Market**						
○ SDID (Main Model)	13.48	0.39	1.50	0.92	-53.55	<0.001
○ DID	15.44	0.37	-2.98	0.84	-55.70	<0.001
○ SC	5.89	0.73	-2.14	0.85	-56.12	<0.001

Note: ATT, average treatment effect on the treated. SDID, synthetic difference-in-differences. DID, traditional difference-in-differences. SC, synthetic control. E-cigarette sales data were obtained from Circana, LLC, point-of-sale (POS) retail scanner data (Multi-Outlet+ and Convenience); Internet and vape shop sales were not available.

[a] E-cigarette directories—state-managed lists of registered products permitted for sale—were published in AL (May 2022), OK (Oct 2023) and LA (Nov 2023).

[b] E-cigarette nicotine sales (mg per capita) were calculated as units sold × e-liquid volume (mL) × nicotine concentration (mg/mL), summed monthly by state, and divided by state population.

[c] Number of distinct products is the number of unique UPCs per month per state.

[d] Covariates include e-cigarette tax per milliliter, unemployment rates, tobacco control funding, state coverage of smoking cessation services, smoke-free laws, and COVID-19 cases.

[e] Effects represent the average treatment effect of directory publication, estimated using synthetic difference-in-differences (SDID) regression for each treated state (AL, OK, LA) compared to its synthetic control. Outcomes were log-transformed; effects expressed as % change: $(e^{\text{SDID coefficient}} - 1) \times 100$. Positive values indicate percentage increases due to directory publication; negative values indicate declines.

[f] States that enacted directory laws but did not publish their lists during the study period (ending April 2025) include Arkansas, Iowa, Mississippi, Nebraska, North Carolina, Pennsylvania, South Carolina, Tennessee, Virginia and Wisconsin.

[g] E-liquid Sales (mL per Capita) were calculated as units sold × e-liquid volume (mL), summed monthly by state, and divided by state population.

© 2026 Ali FRM et al. *JAMA Network Open*.

eTable 4. Overview of State Directory Listings and E-Cigarette Nicotine Sales: April 2025[a,b]

	**Alabama**	**Oklahoma**	**Louisiana**
**Directory Publication Date**	May 1, 2022	October 1, 2023	November 1, 2023
**Total Products Listed**	3,967 (Vape + Alternative Nicotine)	12,219 (Vape)	1,697 ((Vape + Alternative Nicotine))
**Share of Listed E-cigarette Brands from E-cigarette Nicotine Sales per Capita in April 2025 (%)**	7.9	46.4	47.7
**By Product Type**			
**- Disposables (%)**	18.7	39.5	30.8
**- Prefilled Cartridges (%)**	81.3	60.5	69.2
**By Flavor**			
**- Tobacco (%)**	30.1	9.4	12.9
**- Menthol (%)**	54.1	51.4	56.7
**- Mint (%)**	1.8	0.5	10.5
**- Other Flavors (%)**	13.9	38.6	19.8

Note: Authors' compilation based on information from state e-cigarette directories accessed on July 19, 2026 (see text for references). Product classification (listed vs. unlisted) may have overestimated listed-product sales because some products appearing on state directories accessed in July 2026 may not have been listed in April 2025. In addition, coding was conducted at the brand rather than product level, which may have further overestimated listed-product sales if only some products within a brand were directory-eligible. Consequently, estimates of unlisted-product sales are conservative and likely represent a lower bound.

[a] E-cigarette directories—state-managed lists of registered products permitted for sale—were published in AL (May 2022), OK (Oct 2023) and LA (Nov 2023).

[b] E-cigarette sales data were obtained from Circana, LLC, point-of-sale (POS) retail scanner data (Multi-Outlet+ and Convenience); Internet and vape shop sales were not available. E-cigarette nicotine sales (mg per capita) were calculated as units sold × e-liquid volume (mL) × nicotine concentration (mg/mL), summed monthly by state, and divided by state population.

© 2026 Ali FRM et al. *JAMA Network Open*.
