# Supplementary material for: E-Cigarette Directory Laws, Sales, and Product Availability From 3 Early-Adopting US States
Source: JAMA Netw Open. 2026 Sep 15;9(9):e2633874. doi: 10.1001/jamanetworkopen.2026.33874 (PMC13579438; doi:10.1001/jamanetworkopen.2026.33874)
Supplement: Supplementary file 2 [file jamanetwopen-e2633874-s002.pdf]

Data Sharing Statement

Ali. E-Cigarette Directory Laws, Sales, and Product Availability From 3 Early-Adopting US States. *JAMA Netw Open*. Published September 15, 2026.
doi:10.1001/jamanetworkopen.2026.33874

Data
Data available: No

Additional Information
Explanation for why data not available: The retail scanner data used in this study were purchased from Circana, and per our contractual agreement with the company, we do not have the rights to share or publicly distribute these data.
